# Development of a *Tetrahymena thermophila*‐Based Vaccine Expressing *Miamiensis avidus* Ciliary Proteins to Combat Scuticociliatosis

**DOI:** 10.1111/jfd.14097

**Published:** 2025-02-09

**Authors:** Yuho Watanabe, Maho Kotake, Hiromi Matsuoka, Tomoyoshi Yoshinaga, Shin‐Ichi Kitamura

**Affiliations:** ^1^ Department of Aquatic Bioscience, Graduate School of Agricultural and Life Sciences The University of Tokyo Tokyo Japan; ^2^ Graduate School of Bioresources Mie University Tsu Mie Japan

**Keywords:** ciliary protein, *Miamiensis avidus*, scuticociliatosis, *Tetrahymena thermophila*, vaccine

## Abstract

*Miamiensis avidus* is a parasitic pathogen responsible for scuticociliatosis, a lethal infection affecting marine fish worldwide, including Japanese flounder. Immunisation with formalin‐killed *M. avidus* has shown promise in inducing protective immunity, positioning it as a potential vaccine candidate against scuticociliatosis. However, challenges such as the high cost of producing sufficient cells and inconsistent quality due to the lack of cryopreservation methods hinder its development. In this study, we expressed *M. avidus* ciliary proteins in *Tetrahymena*, a culturable ciliate, and used these cells to immunise Japanese flounder. The immunised fish produced antibodies against *M. avidus*. Additionally, immunisation with two transgenic *Tetrahymena* strains, each expressing different ciliary proteins, induced the production of antibodies against two serotypes of the parasite. In challenge experiments, fish immunised with the transgenic *Tetrahymena* showed prolonged survival compared to the control group, highlighting the potential of this approach as a vaccine candidate. These findings suggest that transgenic *Tetrahymena* cells could be a viable platform for developing vaccines against multiple serotypes of *M. avidus*.

## 1 Introduction

*Miamiensis avidus*, a parasitic ciliate, has emerged as a significant pathogen in aquaculture, particularly affecting marine fish species. The parasite was first reported from wild seahorses, indicating its presence in natural marine environments (Thompson and Moewus 1964). The infection caused by this parasite, known as scuticociliatosis, frequently impacts Japanese flounder ( *Paralichthys olivaceus* ), one of the major fish species cultured in countries like Japan and Korea (Song et al. 2009; Narasaki et al. 2018). The parasite invades host tissues, causing lesions, systemic infections and often death (Yoshinaga and Nakazoe 1993; Jung et al. 2007). Due to the high mortality rates associated with *M*. *avidus* infections and the limited effectiveness of current treatments, there is an urgent need for effective preventive measures (Song et al. 2009; Motokawa et al. 2018).

Previous studies have demonstrated that immunisation with formalin‐killed *M. avidus* can induce protective immunity in fish (Jung et al. 2006; Sohn et al. 2023). In other fish‐parasitic ciliates, such as *Ichthyophthirius multifiliis* and *Cryptocaryon irritans*, multiple serotypes exist even within a single species (Dickerson et al. 1993; Misumi et al. 2011). However, research indicates that the serotypes of *M. avidus* present in aquaculture farms are limited (Motokawa et al. 2018), suggesting that immunisation with whole cells may effectively control scuticociliatosis. Despite this, large‐scale cultivation of *M. avidus* for vaccine development is costly and impractical. Moreover, cryopreservation is essential for maintaining antigenicity in formalin‐inactivated vaccines, yet current methods for cryopreserving *M. avidus* are complex and require specialised media and liquid nitrogen (Folgueira et al. 2017).

*Tetrahymena thermophila*, a culturable ciliate widely used as a model organism, offers an alternative approach. Cryopreservation methods for *Tetrahymena* are well‐established (Gaertig and Gorovsky 1992; Cassidy‐Hanley et al. 1997), and it shares the same codon usage as *M. avidus*, making it ideal for expressing recombinant ciliate proteins. Recombinant proteins from *I. multifiliis* and *C. irritans* have been successfully expressed using the *Tetrahymena* system (Gaertig et al. 1999; Mo et al. 2019). Thus, constructing transgenic *Tetrahymena* cells expressing protective antigens from *M. avidus* could pave the way for vaccine development.

In this study, we took the first step towards vaccine development using transgenic *Tetrahymena* expressing surface antigens from two *M. avidus* serotypes common in Japanese aquaculture. We evaluated the antibody production in immunised fish and conducted challenge experiments to assess the protective effects of immunisation with transgenic *Tetrahymena* against infection by *M. avidus*.

## 2 Materials and Methods

### 2.1 Isolates and Maintenance of *M. Avidus*

Two *M. avidus* isolates, JF‐Oi2022 and RSB‐Eh2021, were obtained from Japanese flounder and red sea bream ( *Pagrus major* ), respectively, during disease outbreaks at geographically separated farms in the Oita and Ehime Prefectures, Japan. These isolates were identified as *M. avidus* following the method of Tange et al. (2010). The serotypes of the isolates were determined using agglutination tests with antisera and serotype‐specific PCRs (Motokawa et al. 2018), confirming that JF‐Oi2022 was serotype I and RSB‐Eh2021 was serotype II. The *M. avidus* strains were maintained and subcultured monthly in 25 cm^2^ tissue culture flasks containing P2Y1–1/3 SW medium (2% proteose‐peptone and 1% yeast extract in 10‰ artificial seawater) supplemented with 10% fetal bovine serum (FBS) at 20°C, following Takagishi et al. (2009).

### 2.2 Fish

Naïve fingerlings of *P. olivaceus* were purchased from Marinetech Co. Ltd. (Aichi, Japan), a hatchery that raises fish in uncontaminated saltwater from a saltwater well. Fish from this hatchery had never exhibited *M. avidus* infections in prior purchases. The fish were kept in a 500‐L recirculation tank equipped with a biological filter at 20°C and 30‰ salinity and fed daily with commercial feed (Otohime EP‐2, Marubeni Nissin Feed Co., Tokyo, Japan) until satiation. Skin surfaces, gills, and brain tissue from 10 randomly selected fish were examined under a light and stereo microscope to confirm the absence of *M. avidus* infection. Additionally, experimental fish tested negative for *M. avidus* via agglutination tests with the JF‐Oi2022 and RSB‐Eh2021 isolates.

### 2.3 RNA Extraction and cDNA Synthesis

Total RNA was extracted from each strain of ciliates (1.0 × 10^5^ cells) using a NucleoSpin RNA Plus Kit (Macherey–Nagel, Dürren, Germany), followed by DNase I treatment (Takara Bio, Shiga, Japan) to remove genomic DNA. The RNA was then purified using a NucleoSpin RNA Clean‐up Kit (Macherey–Nagel). Purified RNA (100 ng) was reverse transcribed using the ReverTra Ace qPCR RT Master Mix with gDNA Remover (Toyobo, Osaka, Japan).

### 2.4 Plasmid Construction

**FIGURE 1:**
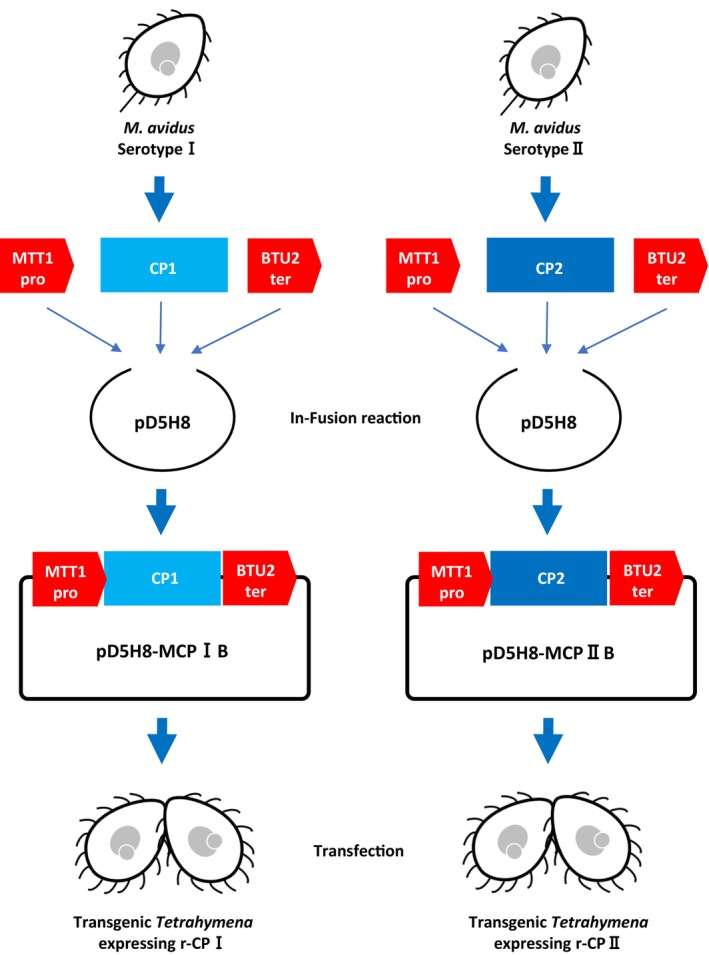
Construction of the pD5H8‐MCP I B and pD5H8‐MCP II B plasmids and generation of transgenic *Tetrahymena* cells expressing r‐CP I and r‐CP II.

The procedure for plasmid construction is shown in Figure 1. To obtain the open reading frame (ORF) sequences of the ciliary protein (CP) from each serotype (LC269180.1 and LC269181.1), RT‐PCR was performed using KOD One PCR Master Mix (Toyobo, Osaka, Japan) with specific primers for each ORF (no. 1 and no. 2 for CPI, no. 3 and no. 4 for CPII, Table 1). The PCR program included 40 cycles of 98°C for 10 s, 50°C for 5 s, and 68°C for 10 s. The cadmium‐inducible MTT1 promoter and beta‐tubulin 2 BTU2 terminator (Shang et al. 2002) were amplified from the Cas9 expression vector pC9T (Tetrahymena Stock Center, Cornell University, Ithaca, NY, USA) (Suhren et al. 2017) using primer pairs no. 5 and no. 6 for the MTT1 promoter of CPI, no. 5 and no. 7 for the MTT1 promoter of CPII, and no. 8 and no. 9 for the BTU2 terminator. The CPI or CPII fragments were cloned into the *Not*I site of the ribosomal DNA vector pD5H8 (Yao and Yao 1989) using an In‐Fusion HD Cloning Kit (Takara Bio), along with the MTT1 promoter and BTU2 terminator fragments. The final plasmids, pD5H8‐MCPIB and pD5H8‐MCPIIB, were sequenced (Eurofins Genomics, Tokyo, Japan) to verify correct insertion. The pD5H8 vector contains *Tetrahymena* MIC rDNA with a paromomycin‐resistant allele of the 17S rRNA (Spangler and Blackburn 1985), facilitating the selection of transformants (Yao and Yao 1989; Taverna et al. 2002). Each plasmid was introduced into *T. thermophila* after transformation into *E. coli* DH5α.

**TABLE 1:** Primers used in this study.

	Primers	Sequence 5'‐3'		Reference
1	CP I_ORF_FW	F	ATGAAAACCGTTATTATCTTAGCTCTTAC	This study
2	CP I_ORF_RV	R	TTTAATTTAAGGATCTCAAATAGCAAAGGAAACCAAAGCG	This study
3	CP II_ORF_FW	F	ATGATTAAATTAATTGTTATAGTAG	This study
4	CP II_ORF_RV	R	TTTAATTTAAGGATCTCAAATGAAGAAGCAGCAATAGC	This study
5	MTT1‐pro_FW	F	CCCGGATCCGCGGCCGCAGACAATTTATTTCTA	Watanabe et al. (2023)
6	MTT1‐pro_RV_CP I	R	AATAACGGTTTTCATTATTTTAAGTTTAGTA	This study
7	MTT1‐pro_RV_CP II	R	AATTAATTTAATCATTATTTTAAGTTTAGTA	This study
8	BTU2‐ter_FW	F	GATCCTTAAATTAAAAATTCAATA	Watanabe et al. (2023)
9	BTU2‐ter_RV	R	GAGAATTCCGCGGCCGCTGCATTTTTCCAGTAAAAATTTG	Watanabe et al. (2023)

### 2.5 *Tetrahymena* Cell Culture and Transformation

Cell culture and transformation were conducted following Watanabe et al. (2023). *Tetrahymena thermophila* strains CU427 (mating type 6) and CU428 (mating type 7) were obtained from the *Tetrahymena* Stock Center (Cornell University) and cultured in NEFF medium (0.25% protease peptone, 0.25% yeast extract, 0.5% glucose, 33 μM FeCl₃) at 30°C for 24 h with shaking (80 rpm). At a density of ~ 5 × 10^5^ cells/mL, 1.0 × 10^7^ cells of each strain were centrifuged at 700 × *g* for 3 min, resuspended in 10 mL of 10 mM Tris–HCl (pH 7.5), and incubated at 30°C for 24 h with shaking (80 rpm). The cells were counted and adjusted to a final density of 2 × 10^5^ cells/mL. Equal numbers of cells from each culture were mixed and incubated for 10 h until more than 80% of the cells had undergone mating. After conjugation, cells were washed twice in 10 mM HEPES (pH 7.5) and concentrated to 3 × 10^7^ cells/mL. Electroporation was performed using 125 μL of the cell suspension and 30 μg of either pD5H8‐MCPIB or pD5H8‐MCPIIB plasmid, using a constant voltage (250 V) for 2.8 ms with a MicroPulser Electroporator (Bio‐Rad Laboratories, Hercules, CA, USA). The cuvettes were rested for 1 min, and the cells were resuspended in 10 mL NEFF medium. Next, 100 μL of NEFF medium was transferred to each well of a 96‐well plate. Eighteen hours post‐electroporation, 100 μL of NEFF containing 200 μg/mL paromomycin sulfate was added to each well, resulting in a final concentration of 100 μg/mL paromomycin sulfate. The plates were incubated at 30°C for 4 days. Wells containing paromomycin‐resistant cells were successfully transferred into NEFF medium with 150, 200 and 400 μg/mL paromomycin to select the final transformed cell lines.

### 2.6 Expression of *M. Avidus* CP in *T. thermophila*

The MTT1 promoter, induced by cadmium ions, regulates CP expression in *T. thermophila* (Shang et al. 2002). Each transgenic *Tetrahymena* cell was cultured in NEFF medium at a density of 1.0 × 10^5^ cells/mL, and CdCl₂ was added to a final concentration of 10 μM for 6 h of induction at 30°C with shaking. Cells were harvested to confirm the expression of *M. avidus* CP.

### 2.7 Indirect Immunofluorescence Antibody Test (IFAT)

IFAT was performed on air‐dried transformed cells fixed in a 1:1 acetone: methanol mixture for 10 min at − 20°C. Cells were immunostained with rabbit antiserum against *M. avidus* serotype I or II at 1:100 dilution in PBS‐T and incubated at 37°C for 60 min, followed by incubation with Alexa Fluor 488‐conjugated donkey anti‐rabbit IgG antibody (1:200) at 37°C for 30 min. Hoechst 33342 solution (1 μg/mL) was used to stain nuclei before fluorescence microscopy (BX60; Olympus, Tokyo, Japan).

### 2.8 Immobilisation/Aggregation Assay of Transgenic *Tetrahymena* Cells

The immobilisation/aggregation assay followed the protocol of Watanabe et al. (2023). Transgenic *Tetrahymena* cells were induced with 10 μM CdCl_2_ for 6 h and exposed to rabbit antiserum (1:100) against *M. avidus* serotypes I and II. Negative controls used uninduced cells. Images were captured with an inverted microscope (Olympus IX71, Tokyo, Japan).

### 2.9 Preparation of Transgenic *Tetrahymena* Cells for Immunisation

Transgenic *Tetrahymena* expressing r‐CPI or r‐CPII were cultured in 500 mL NEFF medium. Cells were formalin‐inactivated at 0.5% (v/v) for 1 h at 4°C, collected by centrifugation (700 × *g*, 3 min), and confirmed inactive by microscopy. Inactivated cells were washed three times with PBS and suspended at 1.0 × 10^6^ cells/mL. Transgenic *Tetrahymena* expressing r‐CPI and r‐CPII were then mixed for immunisation.

### 2.10 Immunisation With Transgenic *Tetrahymena* Cells

A total of 155 fish (mean body weight ± standard deviation [SD]: 11.05 ± 2.09 g) were divided into three groups. To identify the groups, small portions of the pectoral (0.25 cm^2^) and caudal fins (0.5 cm^2^) were clipped. Naïve fish were anaesthetized using 2‐phenoxyethanol (500 ppm) in 10 L of seawater before intra‐peritoneal (IP) injection. Each fish received a 100 μL dose of the vaccine, which contained 2.0 × 10^5^ cells of induced transgenic *Tetrahymena* (50 fish/group). Fish injected with uninduced cells and a group injected with PBS were set as negative controls. The fish were housed together in a 500‐L recirculation tank and fed daily. Before immunisation, blood samples were collected from five fish per group via the hepatic vein using a 1‐mL syringe and a 25‐G needle. The collected blood was left to clot overnight at 4°C, centrifuged at 500 × g for 5 min at 4°C to obtain serum, and stored at −20°C for subsequent enzyme‐linked immunosorbent assays (ELISAs) and agglutination tests to measure antibody production against each *M. avidus* serotype.

Two weeks post‐immunisation, blood was collected from five fish per group for antibody assays, and the remaining 135 fish were boosted with the same concentration of the vaccine (100 μL of 2.0 × 10^5^ cells of transgenic *Tetrahymena*). Fish were maintained in the same conditions as before. Two weeks after the booster, blood was again collected from five fish per group for antibody assays.

For the challenge experiment, methods were adapted from Takagishi et al. (2009) and Shin et al. (2023). Two tanks (150 L, with overhead biofilters, 90 × 45 × 45 cm) were set up with 60 fish per tank at 20°C and 30‰ salinity. After acclimatising for a week, fish were exposed to an *M. avidus* suspension (10^3^ ciliates/mL) in 17‰ seawater (5 L seawater and 5 L dechlorinated water) for 1 h at 20°C. One group of fish was challenged with serotype I *M. avidus* and the other with serotype II. The fish were then returned to the tanks and reared at 20°C without feeding. Ten percent of the water was replaced daily with fresh seawater. Mortality patterns and clinical signs of scuticociliatosis were monitored daily. Dead fish were removed, and *M. avidus* ciliates were observed using wet mount preparations.

### 2.11 ELISAs

ELISAs were conducted following Watanabe et al. (2021) to assess antibody production in the sera of immunised fish. *M. avidus* homogenates from each isolate were diluted to 10 μg/mL in carbonate buffer (0.05 M Na_2_CO_3_ and 0.05 M NaHCO_3_, pH 9.6), and 100 μL of the protein solution was added to the wells of a 96‐well microtiter plate. The plates were incubated overnight at 4°C to allow protein binding. The next day, the plates were blocked with 100 μL of 20% Blocking One (Nacalai Tesque, Kyoto, Japan) in PBS for 1 h at 37°C and washed three times with PBS‐T. Fifty microliters of diluted fish sera (1:100 in 5% Blocking One) was pre‐incubated at 37°C for 1 h to reduce nonspecific antibody reactions, then added to the wells and incubated for 2 h at 37°C. PBS‐T‐washed plates were then treated with 50 μL of anti‐Japanese flounder Ig monoclonal antibody conjugated to horseradish peroxidase (HRP) (1:1000 in 5% Blocking One), incubated for 1 h at 37°C, and washed 10 times. Colour development was induced by adding 100 μL of 3,3,5,5‐tetramethylbenzidine solution (Wako Chemicals, Osaka, Japan), incubating for 30 min at 25°C in the dark, then stopping the reaction with 100 μL of 1 M H_2_SO_4_. Absorbance at 450 nm was measured using a plate reader (iMark; Bio‐Rad Laboratories).

### 2.12 Agglutination Assays

The agglutination assay was conducted following Shin et al. (2023), with minor modifications. Serum samples from fish were incubated at 56°C for 30 min to inactivate the complement system. Serial two‐fold dilutions of the sera were made in Dulbecco's Modified Eagle Medium (DMEM). Freshly harvested *M. avidus* cells were resuspended in DMEM, and their concentration was adjusted using a Burker‐Turk haemocytometer (Hirschmann, Laborgerate Hilgenberg, Germany). Next, the cells were incubated at 15°C for 30 min prior to the agglutination test. Equal volumes (20 μL) of the *M. avidus* suspension (2.0 × 10^3^ cells/well) and diluted sera were added to each well of a 96‐well plate and incubated at 20°C for 3 h. The agglutination or immobilisation reactions were observed every hour for 3 h using a light microscope (Nikon, Japan). The highest dilution showing a positive agglutination reaction was recorded as the titre value for each sample.

### 2.13 Statistical Analysis

Statistical analyses were performed using R software (version 4.4.2) and R studio (version 2024.09.1 + 394). Normality of the data was assessed using the Shapiro–Wilk test, and homoscedasticity was evaluated with Levene's test. Upon confirmation of these assumptions, the results of the ELISA and agglutination assays were analysed using Kruskal‐Wallis tests, followed by Steel‐Dwass post hoc tests. Differences between survival curves were analysed using the log‐rank test with Bonferroni correction for multiple comparisons. Statistical significance was set at *p* < 0.05.

### 2.14 Ethics Statement

Although fish are not covered by national or university animal ethics protocols, the experimental fish were handled in a way that minimised unnecessary pain. To this end, 2‐phenoxyethanol was used for anaesthesia and euthanasia, in line with ethical considerations.

## 3 Results

### 3.1 Expression and Localization of *M. Avidus* Ciliary Proteins in Transgenic *Tetrahymena*

**FIGURE 2:**
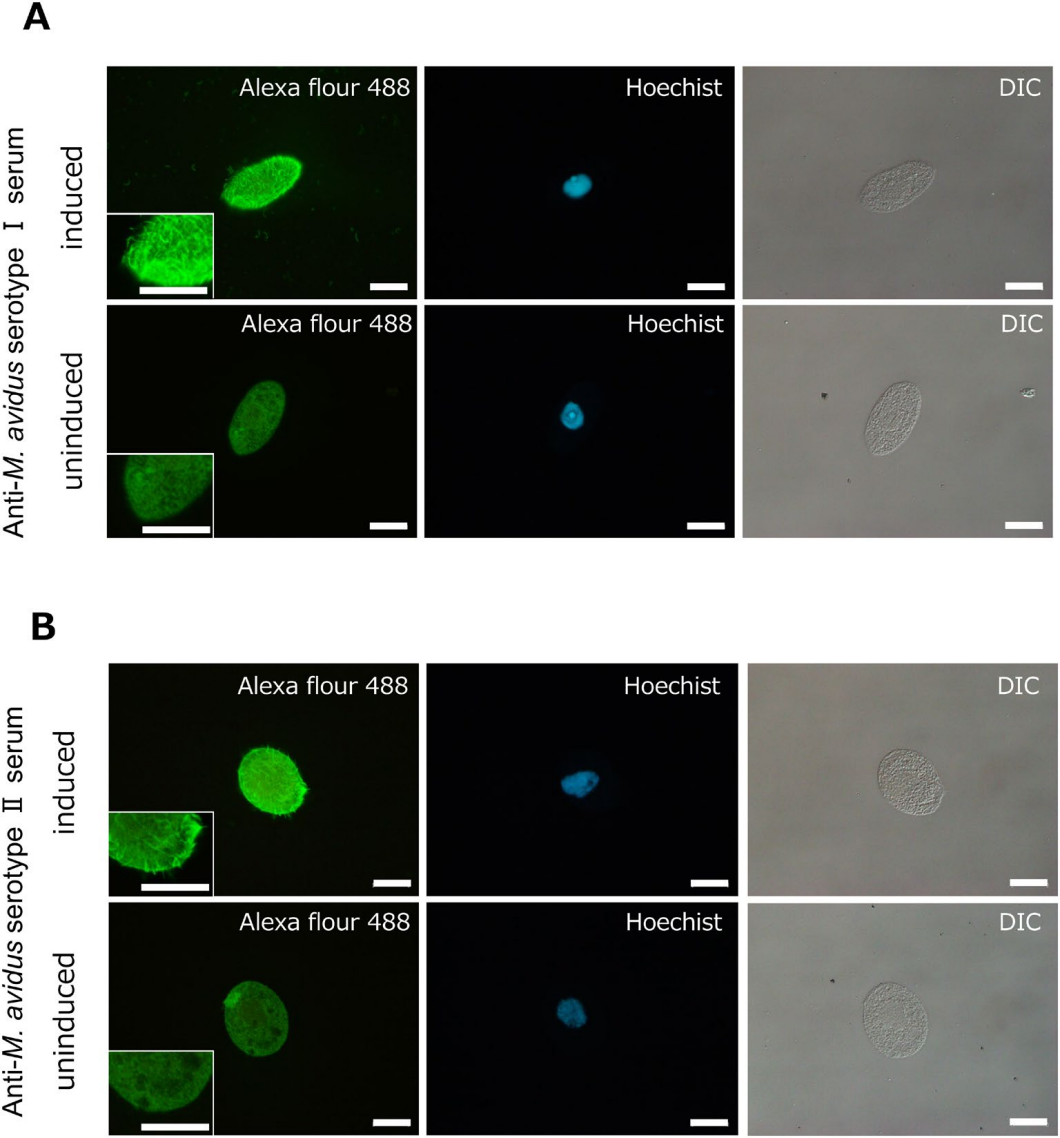
Immunofluorescence antibody test of transgenic *Tetrahymena* cells (pD5H8‐MCPIB, pD5H8‐MCPIIB) using anti‐*M. avidus* serotype I or II serum. Immunofluorescence using anti‐*M. avidus* serotype I (A) and serotype II (B), showing transgenic *Tetrahymena* cells. Scale bar = 20 μm.

Positive clones of each transgenic *Tetrahymena* were successfully obtained after transformation and selection using paromomycin. IFAT with antisera against *M. avidus* serotypes I and II showed strong r‐CPI and r‐CPII signals localised in the cilia (Figure 2). In the uninduced transgenic *Tetrahymena* cells, no signal was detected in the cilia. However, faint signals were visible intracellularly and at the basal part of the cilia. No signal was detected in negative controls treated with normal rabbit serum (Figure S1).

**FIGURE 3:**
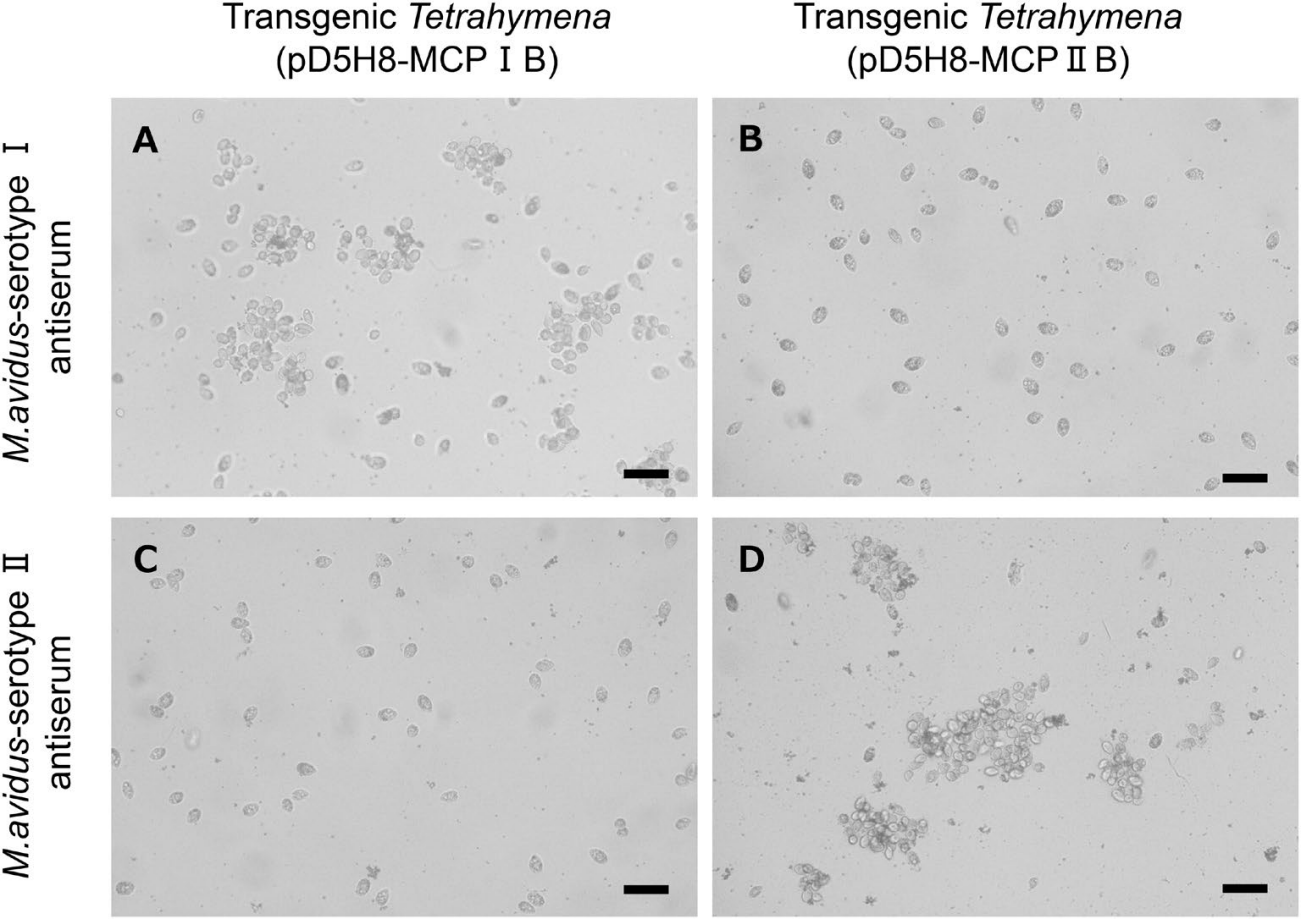
Immobilisation/aggregation assay with transgenic Tetrahymena cells (pD5H8‐MCPIB, pD5H8‐MCPIIB). (A) Expression‐induced transgenic Tetrahymena cells (pD5H8‐MCPIB) incubated with *M. avidus* serotype I antiserum. (B) Expression‐induced transgenic *Tetrahymena* cells (pD5H8‐MCPIIB) incubated with *M. avidus* serotype I antiserum. (C) Expression‐induced transgenic *Tetrahymena* cells (pD5H8‐MCPIB) incubated with *M. avidus* serotype II antiserum. (D) Expression‐induced transgenic *Tetrahymena* cells (pD5H8‐MCPIIB) incubated with *M. avidus* serotype II antiserum. Scale bar = 20 μm.

When the transgenic *Tetrahymena* cells expressing r‐CPI or r‐CPII were incubated with antisera against *M. avidus* serotypes I and II, a serotype‐specific aggregation reaction occurred within 30 min (Figure 3). In contrast, no aggregation or immobilisation was observed in the uninduced cells or negative controls treated with normal rabbit serum (Figure S2).

### 3.2 Antibody Production in Fish Immunised With Transgenic *Tetrahymena*

**FIGURE 4:**
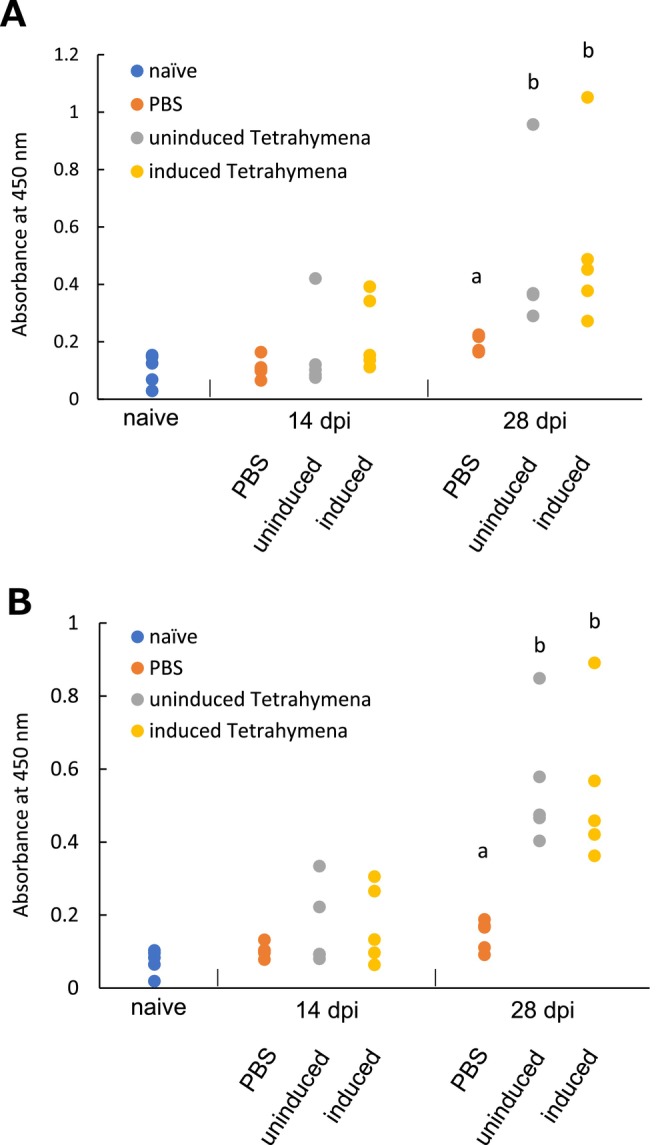
ELISA for antibodies against *M. avidus* serotype I (A) and II (B) in the sera of fish immunised with transgenic *Tetrahymena* cells during the challenge experiment. The graph shows the measurement of specific antibodies by optical absorbance (450 nm) (mean ± SD).

In 28 dpi, sera from fish immunised with induced transgenic *Tetrahymena* cells demonstrated a significant antibody response against both serotypes of *M. avidus* (Figure 4A,B). The antibody titers in these fish were significantly higher than those in naïve fish and fish inoculated with PBS (*p* < 0.05). Fish immunised with uninduced transgenic *Tetrahymena* cells also displayed elevated antibody titers against both serotypes of *M. avidus* compared to naïve fish and fish inoculated with PBS (*p* < 0.05).

### 3.3 Serum Agglutination Titers Against *M. Avidus* in Immunised Fish

**FIGURE 5:**
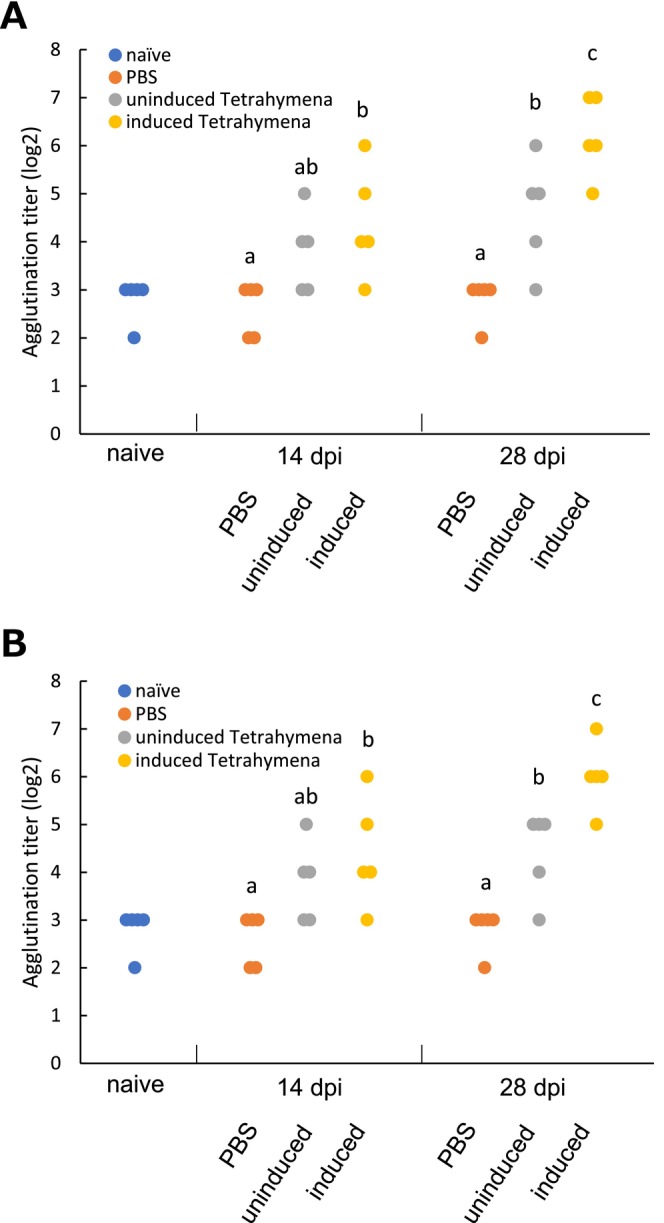
Immobilisation/agglutination tests with *M. avidus* using serum from each group of fish. Immobilisation/agglutination titre of serum from each group of fish against (A) *M. avidus* serotype I and (B) *M. avidus* serotype II. Different lowercase letters denote significant differences in titre (*p* < 0.05).

Fish immunised with transgenic *Tetrahymena* cells exhibited significantly higher agglutination titers against each *M. avidus* serotype compared to the PBS‐inoculated group (Figure 5A,B). Notably, fish immunised with induced transgenic *Tetrahymena* cells showed the highest agglutination titers, while fish inoculated with PBS showed comparable results to naïve fish.

### 3.4 Protective Effects of Transgenic *Tetrahymena* Immunisation in the Survival Challenge

**FIGURE 6:**
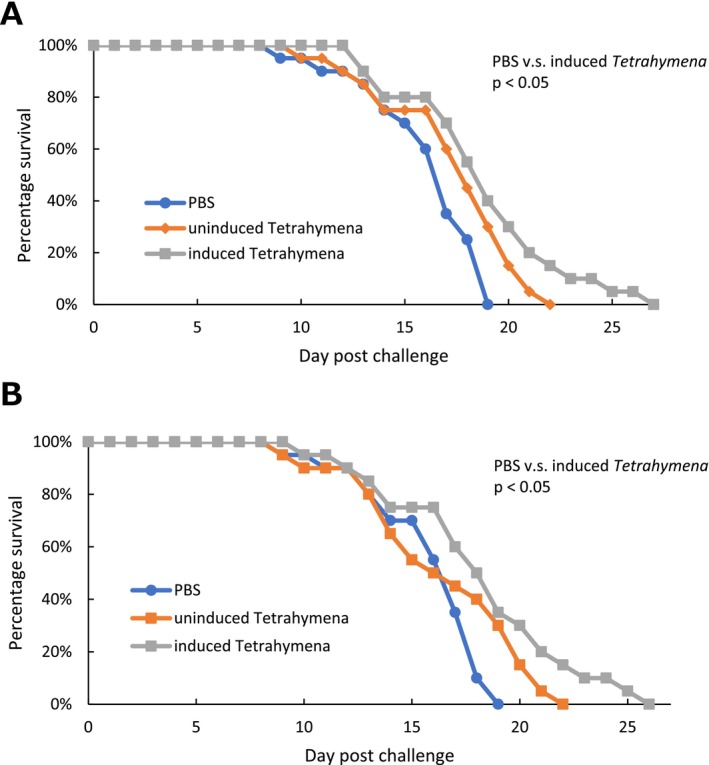
Survival curves from challenge experiments showing cumulative mortality in fish injected with induced transgenic *Tetrahymena* cells, uninduced transgenic *Tetrahymena* cells, and the PBS control group. Survival curves from the challenge experiment using (A) *M. avidus* serotype I and (B) *M. avidus* serotype II. Significant differences in survival time were observed only in the groups injected with induced transgenic Tetrahymena cells, compared to the PBS‐injected group, in both trials (*p* < 0.05).

In the challenge experiment using *M. avidus* serotype I (Figure 6A), although all experimental fish eventually died, the group injected with transgenic *Tetrahymena* cells tended to have a longer survival period compared to the PBS‐injected group. Furthermore, the group injected with induced transgenic *Tetrahymena* cells showed a significant difference in survival time compared to the PBS‐injected group (*p* < 0.05).

Similarly, in the challenge test using serotype II (Figure 6B), all experimental fish died. However, the group injected with transgenic *Tetrahymena* cells tended to have a longer survival period and the group injected with induced transgenic *Tetrahymena* cells showed a significant difference in survival time compared to the PBS‐injected group (*p* < 0.05).

Parasite infection was confirmed in all dead fish, regardless of group.

## 4 Discussion

Immunisation using *M. avidus* cells has been shown to reduce mortality from scuticociliatosis (Jung et al. 2006; Sohn et al. 2023). Recent advancements in oral administration methods further suggest the potential for a vaccine targeting this parasitic infection (Shin et al. 2023). However, there are significant challenges, including the high cost of producing sufficient cells for vaccine development and the complexity of cryopreservation, which impedes commercial scalability. In this study, we successfully expressed *M. avidus* CPs in *Tetrahymena*, leading to antibody production in immunised fish. The results suggest that transgenic *Tetrahymena* could be a promising candidate for vaccine development against *M. avidus*. Unlike *M. avidus*, *Tetrahymena* is easy to mass‐produce and cryopreserve, making it a cost‐effective solution for overcoming production‐related challenges.

Notably, weak signals were observed in IFAT with antisera against *M. avidus* in uninduced transgenic *Tetrahymena*. Fish immunised with uninduced transgenic *Tetrahymena* also showed elevated antibody and agglutination titers, suggesting the presence of conserved antigens between *Tetrahymena* and *M. avidus*. This finding is consistent with previous reports of conserved antigens shared between *Tetrahymena* and *Cryptocaryon irritans* (Mo et al. 2022), underscoring *Tetrahymena's* potential as a platform for parasitic ciliate vaccines.

Although the challenge experiments showed prolonged survival in immunised fish, all fish eventually died, indicating that the current vaccine formulation provided only partial protection. Previous studies using *M. avidus* cell vaccines administered 5 × 10^5^ cells/fish (Sohn et al. 2023), suggesting that a higher cell count might be necessary to achieve more robust protection. It is also possible that the quantity of ciliary proteins expressed by the recombinant *Tetrahymena* in this study differed from that of *M. avidus*, potentially contributing to the limited protective effect observed. Moreover, research shows that adjuvants can improve the protective effects of *M. avidus* vaccines (Jung and Jung 2021), highlighting the need for further studies on vaccine formulation and administration methods.

While the vaccine extended survival time, all fish eventually showed clinical signs of scuticociliatosis, indicating that the vaccine did not prevent tissue invasion by *M. avidus*. Successful infection prevention often requires both local and systemic immunity (Somamoto and Nakanishi 2020). The IP injection method used in this study likely induced systemic immunity but did not confer local mucosal immunity, which is essential for preventing *M. avidus* from establishing infection at the site of entry. Future research should explore alternative vaccination methods, such as immersion or stamp vaccination, to induce mucosal immunity. Recent studies using chitosan microsphere‐encapsulated inactivated vaccines have successfully induced antibodies in both serum and mucus, demonstrating protective effects against scuticociliatosis (Shin et al. 2023). Combining this approach with transgenic *Tetrahymena* vaccines could lead to a more effective strategy for preventing *M. avidus* infections.

## 5 Conclusions

This study demonstrated that transgenic *Tetrahymena* expressing the CP of *M. avidus* successfully induced antibody production and provided partial protection against parasitic infection. Immunisation with two different transgenic *Tetrahymena* strains expressing distinct CPs also induced antibodies against both serotypes of *M. avidus*, suggesting the potential for a multi‐serotype vaccine. Furthermore, these findings indicate that the ciliary protein (CP) from *M. avidus* represents a promising candidate as a target antigen for vaccine formulation.

However, despite these promising results, the current formulation provided limited protection, and further research is necessary to improve its efficacy. In addition, there are challenges to address for practical application. Although this study did not extensively examine these aspects, previous studies have suggested some factors such as the amount of antigen administered, delivery methods, the use of adjuvants, and environmental conditions like water temperature, which can influence vaccine efficacy (Nakanishi 1983; Anderson 1996; Sommerset et al. 2005). Comprehensive research integrating these factors, including conditions that maximise protective effects, is necessary to establish recommended protocols for vaccine use.

The transgenic *Tetrahymena* system offers a novel platform for generating immunogenic mimics of parasitic ciliates, with significant potential for vaccine development against *M. avidus* and other related pathogens.

## Author Contributions

**Yuho Watanabe:** conceptualization, investigation, writing – original draft. **Maho Kotake:** investigation. **Hiromi Matsuoka:** investigation. **Tomoyoshi Yoshinaga:** supervision. **Shin‐Ichi Kitamura:** conceptualization, writing – review and editing, supervision.

## Conflicts of Interest

The authors declare no conflicts of interest.

## Supporting information


Data S1.

## Data Availability

The data that support the findings of this study are available from the corresponding author upon reasonable request.
