# Supplementary material for: Development of a Tetrahymena thermophila‐Based Vaccine Expressing Miamiensis avidus Ciliary Proteins to Combat Scuticociliatosis
Source: J Fish Dis. 2025 Feb 9;48(6):e14097. doi: 10.1111/jfd.14097 (PMC12068840; doi:10.1111/jfd.14097)
Supplement: Supplementary file 1 — Data S1. [file JFD-48-e14097-s001.pdf]

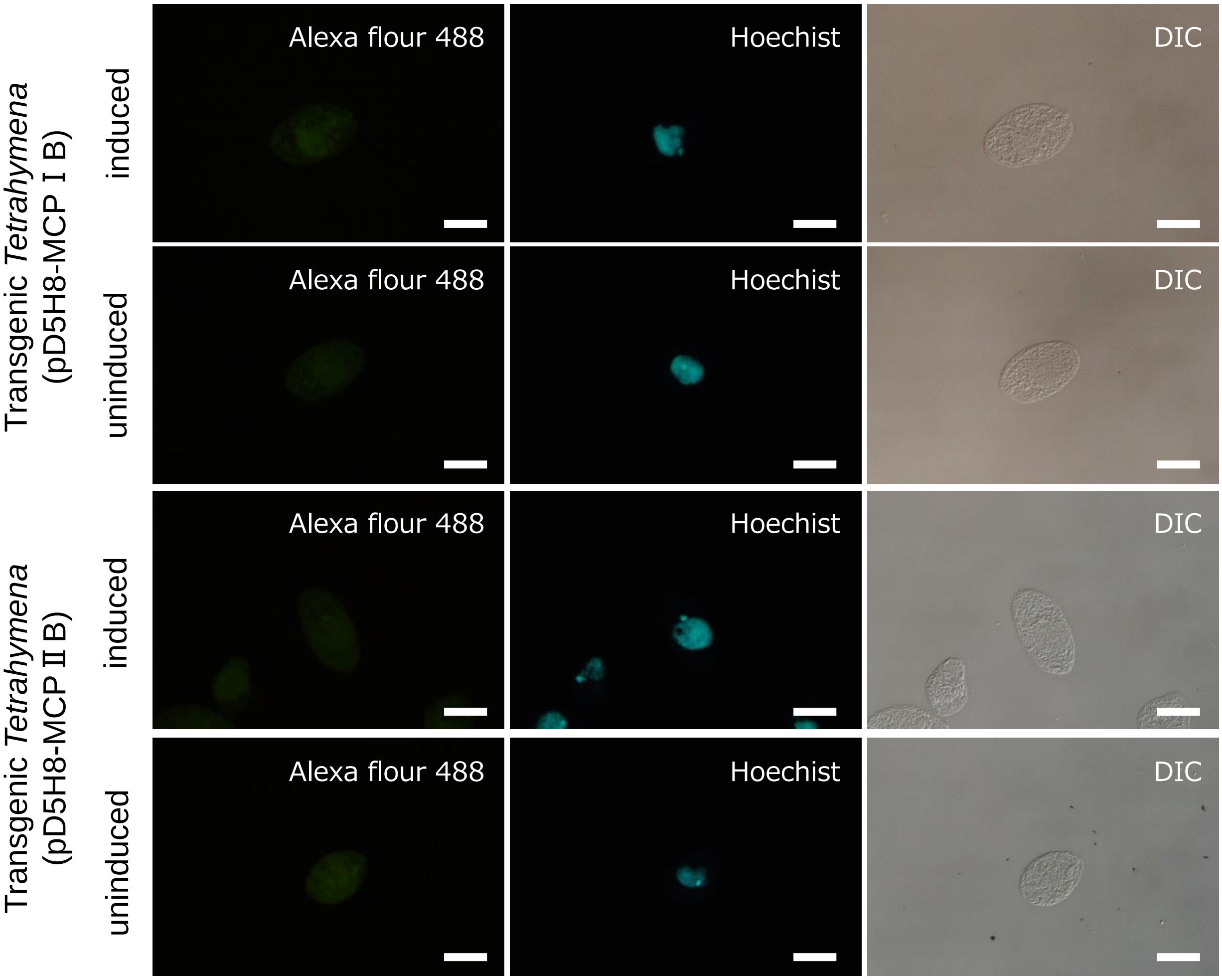

Fig. S1 Immunofluorescence antibody test of transgenic *Tetrahymena* cells (pD5H8-MCPIB, pD5H8-MCPIIB) using normal rabbit serum. Scale bar = 20 μm.

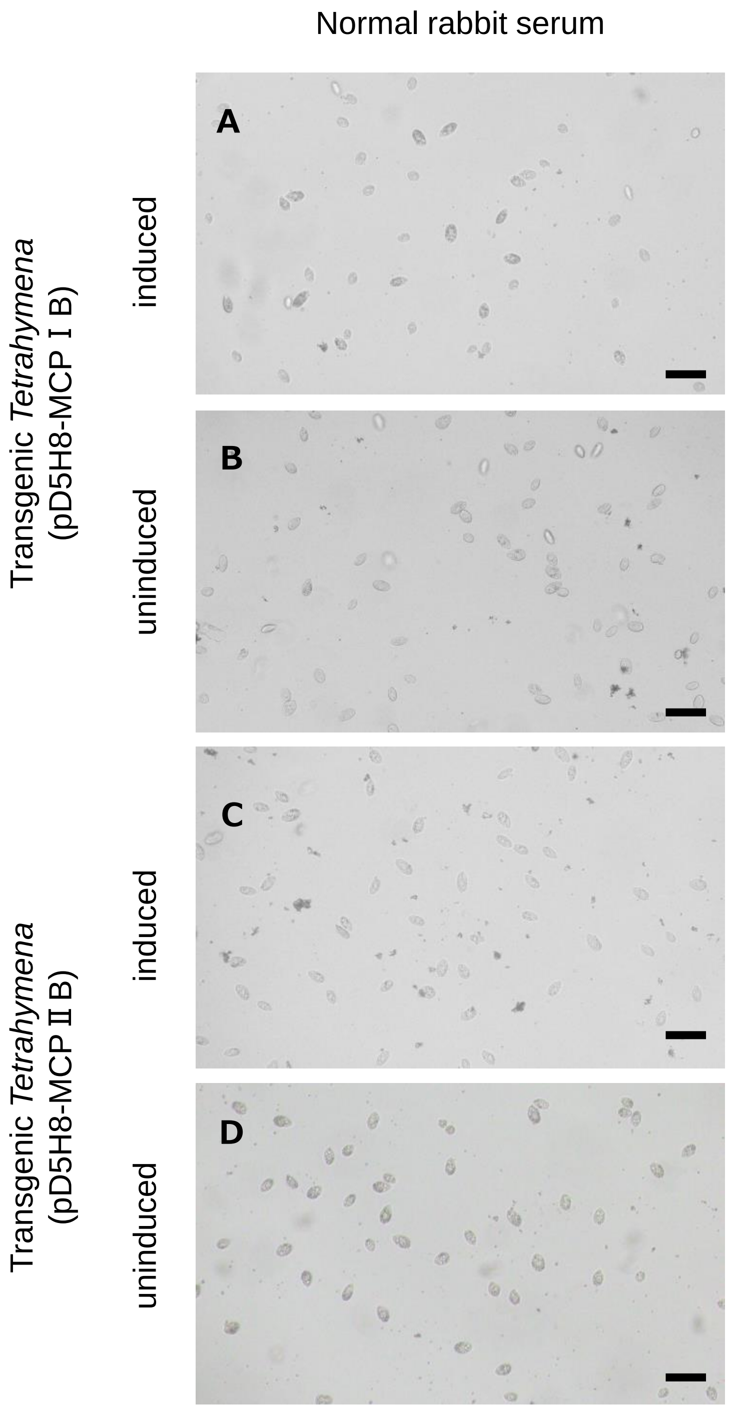

Fig. S2 Immobilization/aggregation assay of transgenic *Tetrahymena* cells incubated with normal rabbit serum. Expression-induced (A) or uninduced (B) transgenic *Tetrahymena* cells (pD5H8-MCPIB) incubated with normal rabbit serum. Expression-induced (C) or uninduced (D) transgenic *Tetrahymena* cells (pD5H8-MCPIIB) incubated with normal rabbit serum. Scale bar = 20 μm.
